# Incidence and risk factors of hypertension therapy in Australian cancer patients treated with vascular signalling pathway inhibitors

**DOI:** 10.1007/s12672-022-00468-3

**Published:** 2022-01-20

**Authors:** Soojung Hong, Benjamin Daniels, Marina T. van Leeuwen, Sallie-Anne Pearson, Claire M. Vajdic

**Affiliations:** 1 Centre for Big Data Research in Health, UNSW Sydney, Sydney, Australia; 2 Division of Oncology-Hematology, Department of Internal Medicine, National Health Insurance Service, Ilsan Hospital, Ilsan-ro 100, Goyang, Republic of Korea

**Keywords:** Cancer, Hypertension, Vascular signalling pathway inhibitors, Incidence, Risk factors

## Abstract

**Background:**

Clinical trials report systemic hypertension is an adverse effect of vascular signalling pathway inhibitor (VSPi) use. There are limited data from routine clinical practice. We aimed to estimate the real-world incidence and risk factors of new-onset and aggravated hypertension for cancer patients dispensed VSPi in whole-of-population Australian setting.

**Methods:**

We used dispensing records for a 10% random sample of Australians to identify treatment with subsidised VSPi from 2013 to 2018. We further identified dispensings of oral antihypertensive medicines 6 months before and 12 months after VSPi therapy. We defined (i) new-onset hypertension in people first dispensed antihypertensives after VSPi and (ii) aggravated hypertension in people with prior antihypertensive use dispensed an additional, or higher strength, antihypertensive after VSPi. We applied the Fine-Gray cumulative incidence function and Cox proportional hazard regression.

**Results:**

1802 patients were dispensed at least one VSPi. The mean age of the cohort was 65 years and 57% were male. The incidence of new-onset treated hypertension was 24.3% (95%CI: 21.2–27.8); age ≥ 60 years (HR 1.74; 95%CI: 1.32–2.31) and treatment with oral tyrosine kinase inhibitors compared to bevacizumab (HR 1.96; 95%CI: 1.16–3.31) were risk factors. The incidence of aggravated hypertension was 25.2% (95%CI: 22.0–28.7) and risk was elevated for patients with renal cancer (HR 2.84; 95%CI: 1.49–5.41) and cancers other than colorectal (HR 1.85; 95%CI: 1.12–3.03).

**Conclusions:**

Our real-world estimates of incident hypertension appear comparable to those observed in clinical trials (21.6–23.6%). Our population-based study provides some insight into the burden of hypertension in patients commencing VSPi in routine practice.

## Introduction

Angiogenesis has an important role in tumour cell proliferation and metastasis, making the vascular endothelial growth factor (VEGF) signalling pathway (VSP) a critical therapeutic target of cancer therapy [1]. Several medicines targeting the VSP are available for cancer treatment and VSP inhibitors (VSPi) are currently one of the most common medicine classes used in the treatment of solid cancers [2, 3]. These agents also target normal cardiovascular physiology, known as an ‘on-target’ effect [4]. This can lead to adverse cardiovascular effects including hypertension, thrombosis, heart failure and stroke [5, 6]. Systemic hypertension, the most common cardiotoxicity in patients using VSPi, results from increased vascular resistance and systemic thrombotic microangiopathy [7]. The incidence of hypertension associated with bevacizumab, the most common VSPi, is 23.6% in clinical trials [8–10]. The incidence of VSPi-induced hypertension from other meta-analyses range from 21.6% with sunitinib [11] to 47% with lenvatinib [12]. If not properly managed, hypertension can cause end organ damage to the heart, kidney, and brain [13]. In addition, if it is not controlled or complications occur, the VSPi could be discontinued, potentially impairing cancer control and shortening patient survival.

All meta-estimates for VSPi-induced hypertension are based on phase II/III clinical trials, where patients are generally highly selected, younger and with fewer comorbidities than typical cancer patients. Currently, there are limited real-world data and no whole-of-population estimates of new onset and aggravated hypertension in VSPi treated patients. A United States’ (US) claims-based study of commercially insured patients reported new-onset hypertension in 32% of a cohort of approximately 1000 cancer patients receiving VSPi [14]. Another US observational study based on electronic medical records from a single health care network found 50% of approximately 1100 patients had VSPi-induced hypertension (new onset or aggravated), with elevated risk for those with pre-existing hypertension, age ≥ 60 years, and higher body mass index [15]. Both of these studies defined hypertension occurrence as a diagnostic code for hypertension or a pharmacy claim for an antihypertensive. Here we describe the real-world incidence and risk factors of new-onset and aggravated hypertension for patients dispensed VSPi in a whole-of-population setting in Australia.

## Methods

### Study design

A population-based, retrospective cohort study.

### Data source and study population

All Australian citizens and permanent residents are entitled to subsidised prescribed medicines through the Pharmaceutical Benefit Scheme (PBS). We used dispensing records for a 10% random sample of PBS-eligible Australians. The 10% PBS sample is a routine dataset provided by Services Australia for analytical use; people are selected based on the last digit of a randomly assigned unique identifier. This dataset captures all dispensed PBS-listed medicines, including PBS medicines not attracting a subsidy, from 1 July 2012 [16]. To protect the privacy of people in this dataset, all dates of dispensing are offset randomly by ± 14 days; the direction of the offset is the same for all records for each individual [17].

Our study population included all adults (aged ≥ 18 years) with a new dispensing record for a VSPi between 1 January 2013 and 30 September 2018. We defined the index treatment date as the first date of VSPi dispensing in this period. Allowing for a 180 day look-back period and at least one year follow-up, the study period was dispensing records from 1 July 2012 to 30 September 2019. We excluded patients with less than one-month follow-up and those with a single dispensing claim.

### Medicines of interest

We identified all PBS-subsidised VSPi medicines in our sample; bevacizumab, sunitinib, sorafenib, pazopanib, axitinib and lenvatinib. Bevacizumab is a monoclonal antibody administered intravenously on a 2- or 3-week schedule for colorectal (CRC), cervical, and ovarian cancer. The other agents are small molecule tyrosine kinase inhibitors (TKIs) administered daily orally, with one prescription lasting 30 days. In Australia, sunitinib is indicated for pancreatic neuroendocrine tumours (pNET), malignant gastrointestinal stromal tumours (GIST), and renal cell carcinoma (RCC). Sorafenib is indicated for RCC and hepatocellular carcinoma (HCC), pazopanib for RCC and soft tissue sarcoma (STS), axitinib for RCC, and lenvatinib for thyroid cancer [18]. We determined primary cancer site based on the VSPi PBS item code dispensed to each patient.

We classified dispensings of PBS-listed oral antihypertensive medicines using the Anatomical Therapeutic Chemical (ATC) Classification System: C03 Diuretics, C07 Beta-blockers, C08 Calcium channel blockers, and C09 Agents acting on the renin-angiotensin system including fixed-dose combinations. We excluded the C02 (antihypertensives) class because these treatments are more commonly used to treat conditions other than hypertension in Australia [19]. The dispensing of hypertensive medications was used to identify pre-existing hypertension and also used for our outcome measure.

### Measures

We calculated the duration of oral VSPi medicine as the first to the last dispensing date plus the dispensed quantity (30 days). For bevacizumab, the duration of use was calculated as the first to the last dispensing date plus 14 days because more than 90% of patients received this medicine every 2-weeks.

We classified people with at least one antihypertensive dispensing record in the 180 days prior to the date of first VSPi dispensing as having pre-existing pharmacologically treated hypertension. All other patients were classified as having no prior hypertension treatment. We defined new-onset hypertension as people first dispensed an antihypertensive whilst being dispensed a VSPi. We defined the aggravated hypertension group as those patients with pre-existing hypertension who were dispensed an additional, or higher tablet strength (escalated dose same or replacement/switched agent), antihypertensive whilst being dispensed a VSPi. We identified other comorbidities (excluding malignancy and hypertension) using the Rx-risk comorbidity score applied to dispensing records for the six months prior to commencing VSPi treatment [20]. The Rx-risk is a measure of an individual’s current comorbidities based on their dispensing history. It has been shown to predict 1-year mortality in multiple prescription datasets, including the 10% PBS sample [20]. Each identified comorbidity was given a score of one, and the scores were summed for each individual to derive an overall comorbidities score.

### Statistical analysis

All patients were followed from the index date until the first dispensing or change in antihypertensive medicine, the end of VSPi treatment, death, or the end of follow up, whichever came first.

We defined the incidence rate as the number of patients who developed new onset or aggravated hypertension during follow-up divided by the total person-time of observation. We presented these as events per 1000 person-years and 95% confidence intervals (95%CI). We considered death as a competing risk for the time to event analyses. The dataset provided the fact and year of death; we defined the date of death as the last date of any dispensed medicines plus 90 days [21]. We calculated the incidence of hypertension (new onset and aggravated) overall and by sex, age, comorbidity, type of cancer, and type of VSPi using the Fine and Gray cumulative incidence function [22].

We compared the baseline characteristics of people with ‘no prior treated hypertension’ and ‘pre-existing treated hypertension’ using the Chi-square test. We described the prescribing pattern of antihypertensive medicines including the first-line therapy and the number of antihypertensive medicines. We compared the median time from VSPi dispensing to first dispensing of an antihypertensive agent for patients treated with oral TKIs and bevacizumab using quantile regression. To evaluate risk factors for developing new onset or aggravated hypertension, we used cause-specific Cox proportional hazard regression to estimate hazard ratios (HR) and 95%CIs. We examined the association with all available putative risk factors, specifically age, sex, comorbidity, cancer type, and type of VSPi. All statistical analyses were conducted using SAS version 9.4 (SAS Institute, Cary NC).

## Results

### Cohort characteristics

**Fig. 1:**
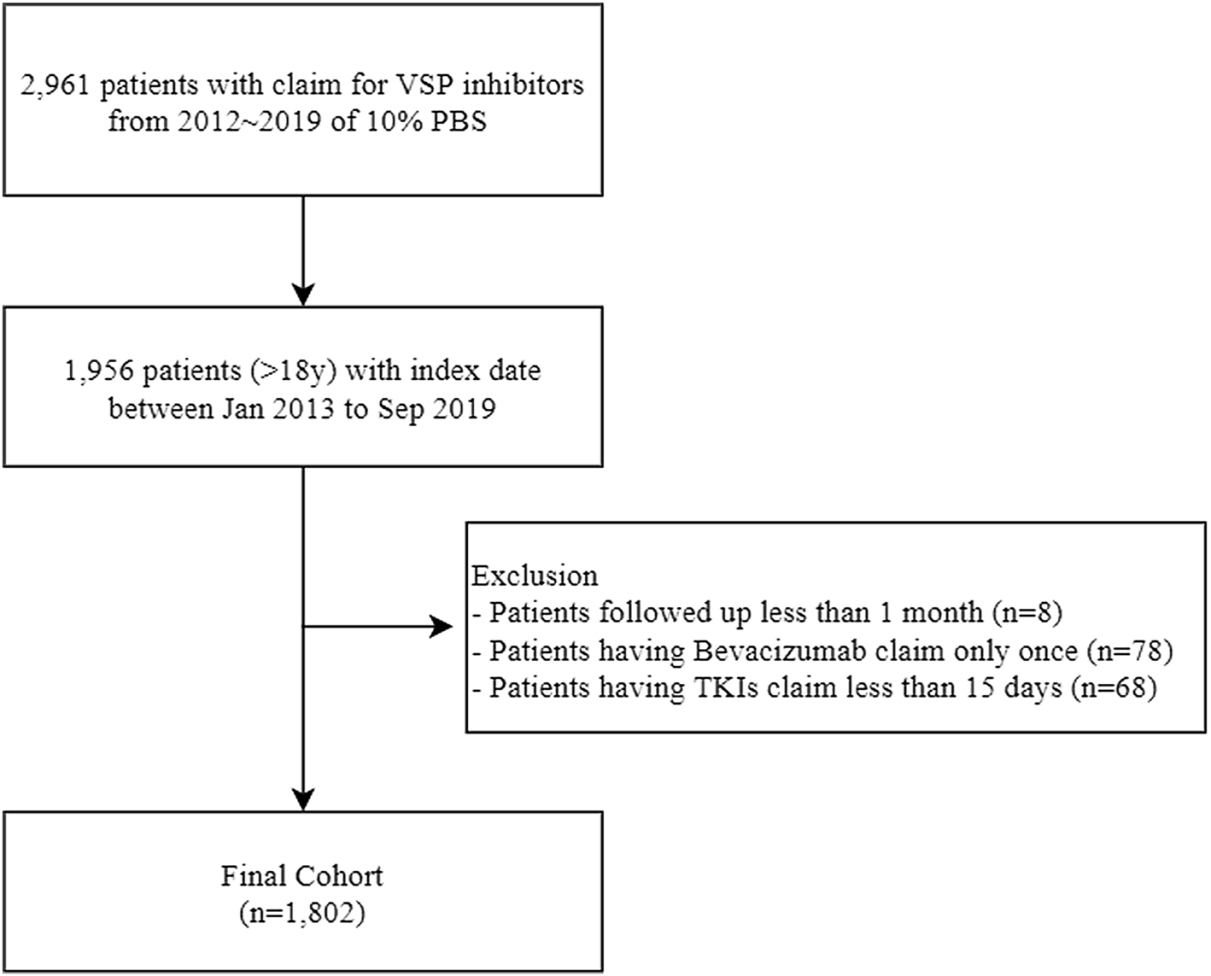
Consort flow diagram of study population

We identified 1802 patients fulfilling our criteria for VSPi use (Fig. 1), 906 (50.3%) of whom had pre-existing treated hypertension. The mean age of the cohort was 65.1 years (SD 12.5); people with pre-existing treated hypertension (66.0 years) were older than those with no prior hypertension (60.1 years) (Table 1). Overall, there were more males than females (57.5% vs. 42.5%), and a higher proportion of males with the pre-existing hypertension. The mean comorbidity score was 3.2 (SD 2.1) for all patients, and the comorbidity burden was higher in the pre-existing compared to the no prior hypertension group. The most common cancer was CRC (58.2%), followed by RCC (14.8%) and HCC (12.3%).

**Table 1:** Study population dispensed VSPi (n = 1,802)

Characteristic	Overall (n = 1802)		No prior HTN (n = 896)		Prior HTN (n = 906)		P-value
	n	%	n	%	n	%	
Mean age (years)	65.1 (SD 12.5)		60.1 (SD 12.7)		66.0 (SD 10.2)		< 0.01
Sex							
Male	1,036	57.5	470	52.5	566	62.5	< 0.01
Female	766	42.5	426	47.5	340	37.5	
Number of comorbid conditions (Rx-risk)							
Mean	3.2 (SD 2.1)		2.5 (SD 1.8)		3.9 (SD 2.2)		< 0.01
0	143	7.9	116	13.0	27	3.0	< 0.01
1–2	591	32.8	366	40.9	225	24.8	
3–4	611	33.9	288	32.1	323	35.7	
≥ 5	457	25.4	126	14.1	331	36.5	
Cancer type							
Colorectal	1,049	58.2	560	62.5	489	54.0	< 0.01
Renal	267	14.8	114	12.7	153	16.9	
Liver	222	12.3	60	6.7	162	17.9	
Ovarian	134	7.4	84	9.4	50	5.5	
Sarcoma	55	3.1	36	4.0	19	2.1	
GIST	26	1.4	14	1.6	12	1.3	
Thyroid	20	1.1	7	0.8	13	1.4	
Cervix	20	1.1	16	1.6	4	0.4	
Pancreatic NET	9	0.5	5	0.6	4	0.4	
VSPi agent							
Bevacizumab	1,203	66.8	660	73.7	543	59.9	< 0.01
Sorafenib	234	13.0	66	7.4	168	18.5	
Pazopanib	218	12.1	104	11.6	114	12.6	
Sunitinib	126	7.0	59	6.6	67	7.4	
Lenvatinib	20	1.1	7	0.8	13	1.4	
Axitinib	^a^	0	^a^	0	^a^	0.1	
Prior chemotherapy (6 months prior to VSPi)							
No	881	48.9	394	44.03	487	53.8	< 0.01
Yes	921	51.1	502	56.0	419	46.3	
Type of prior chemotherapy							
Fluorouracil	725	44.4	376	41.5	349	48.0	0.02
Platinum	526	32.2	289	31.9	237	32.6	
Irinotecan	125	7.7	75	8.3	50	6.9	
Taxane	110	6.7	71	7.8	39	5.4	
Anti-EGFR Ab	38	2.3	25	2.8	13	1.8	
Anthracycline	35	2.1	25	2.8	10	1.4	
Other	75	4.6	46	5.1	29	4.0	

*EGFR* epidermal growth factor receptor, *Ab* antibody, *HTN* hypertension, *GIST* gastrointestinal stromal tumour, *NET* neuroendocrine tumour

^a^Less than 3; suppressed to protect patient confidentiality

Bevacizumab was the most frequently used VSPi (66.8% of all patients), and the rate of use was higher in people who had not been treated previously for hypertension compared with those previously treated (73.7% vs 59.9%). Among oral TKIs, the most frequently prescribed medicine was sorafenib in people who had pre-existing hypertension and pazopanib in people who had no prior hypertension. About half the patients (48.9%) were dispensed VSPi as the first line treatment for this therapeutic stage of disease. The remainder most frequently received fluorouracil (5-FU and capecitabine) and platinum (cisplatin, carboplatin, and oxaliplatin) classes in the 6 months prior to using VSPi. Patients were treated with VSPi for a median of 206 days (range 15–2176) and the duration was not significantly different between the two groups (226 days in no prior hypertension group vs. 197 days in pre-existing hypertension group).

### Hypertension incidence

The total duration of follow-up was 1194 person-years, and the average was 0.66 person-years (SD 0.79) or 241 days. For the 896 people without a history of treated hypertension, new-onset hypertension occurred during VSPi use in 218 patients (Table 2). The overall cumulative incidence over 1 year was 24.3% (95% CI, 21.2–27.8) and the incidence rate was 352.8/1000 person-years (95% CI, 308.9–402.9). The median time from VSPi dispensing to first dispensing of an antihypertensive agent was 78 days (range 4–945); significantly shorter for patients treated with oral TKIs than bevacizumab (median 53 vs 95 days). Most (49%) people with new-onset hypertension were initially prescribed a single antihypertensive agent; most commonly angiotensin-converting enzyme (ACE) inhibitors or angiotensin receptor blockers (ARBs) (47.8%, N = 106). Fifty-nine patients (26.6%) initially received a calcium channel blocker (CCB); only six patients were dispensed combination agents at the time of treatment initiation.

**Table 2:** Characteristics of VSPi cohort subgroups with and without pre-existing hypertension

Characteristic	No previous hypertension					Pre-existing hypertension				
	No incident hypertension (n = 678)		New-onset hypertension (n = 218)		P-value	No aggravated hypertension (n = 678)		Aggravated hypertension (n = 228)		P-value
	n	%	n	%		n	%	n	%	
Mean age	59.04 (SD 12.98)		63.36 (SD 10.98)		< 0.01	69.72 (SD 10.42)		70.66 (SD 9.66)		0.24
Sex										
Male	352	51.9	118	54.1	0.57	431	63.6	135	59.21	0.43
Female	326	48.1	100	45.9		247	36.4	93	40.79	
Number of comorbid conditions (Rx-Risk)										
Mean	2.5 (SD 1.8)		2.4 (SD 1.8)		0.45	4.0 (SD 2.2)		3.8 (SD 2.2)		0.23
0	85	12.5	31	14.2	0.66	19	2.8	8	3.5	0.26
1–2	275	40.6	91	41.7		158	23.3	67	29.4	
3–4	225	33.2	63	28.9		249	36.7	74	32.5	
≥ 5	93	13.7	33	15.1		252	37.2	79	34.7	
Cancer type										
Colorectal	447	65.9	113	51.8	< 0.01	377	55.6	112	49.1	0.09
Renal	74	10.9	40	18.4		104	15.3	49	21.5	
Liver	39	5.8	21	9.6		127	18.7	35	15.4	
Ovarian	59	8.7	25	11.5		31	4.6	19	8.3	
Sarcoma	28	4.1	8	3.7		15	2.2	4	1.8	
GIST	6	0.9	8	3.7		9	1.3	3	1.3	
Others	25	3.7	3	1.4		15	2.2	6	2.6	
VSPi										
Bevacizumab	521	76.8	139	63.8	< 0.01	411	60.6	132	57.9	0.47
TKI	157	23.2	79	36.2		267	39.4	96	42.1	

*GIST* gastrointestinal stromal tumour, *TKI* tyrosine kinase inhibitor

A total of 906 patients had hypertension prior to VSPi therapy. ACE inhibitors or ARB were the most frequently prescribed (32.4%), followed by combination agents (19.1%), beta-blockers (18.1%), CCB (15.3%), and diuretics (15.1%). Among them, 228 patients experienced aggravated hypertension requiring an additional, or higher tablet strength, antihypertensive. The overall cumulative incidence of aggravated hypertension over 1 year was 25.2% (95% CI, 22.0–28.6) and the incidence rate was 395.6/1000 person-years (95%CI, 347.5–450.4). The median time from the first VSPi dispensing to aggravation was 70 days (range 2–1806). Patients with aggravated hypertension were dispensed an average of two antihypertensive agents before aggravation and an average of three agents after aggravation. After aggravation, ACE inhibitors, ARBs and combination ACE/ARB agents were dispensed less often, and CCBs and diuretics were dispensed more often.

**Fig. 2:**
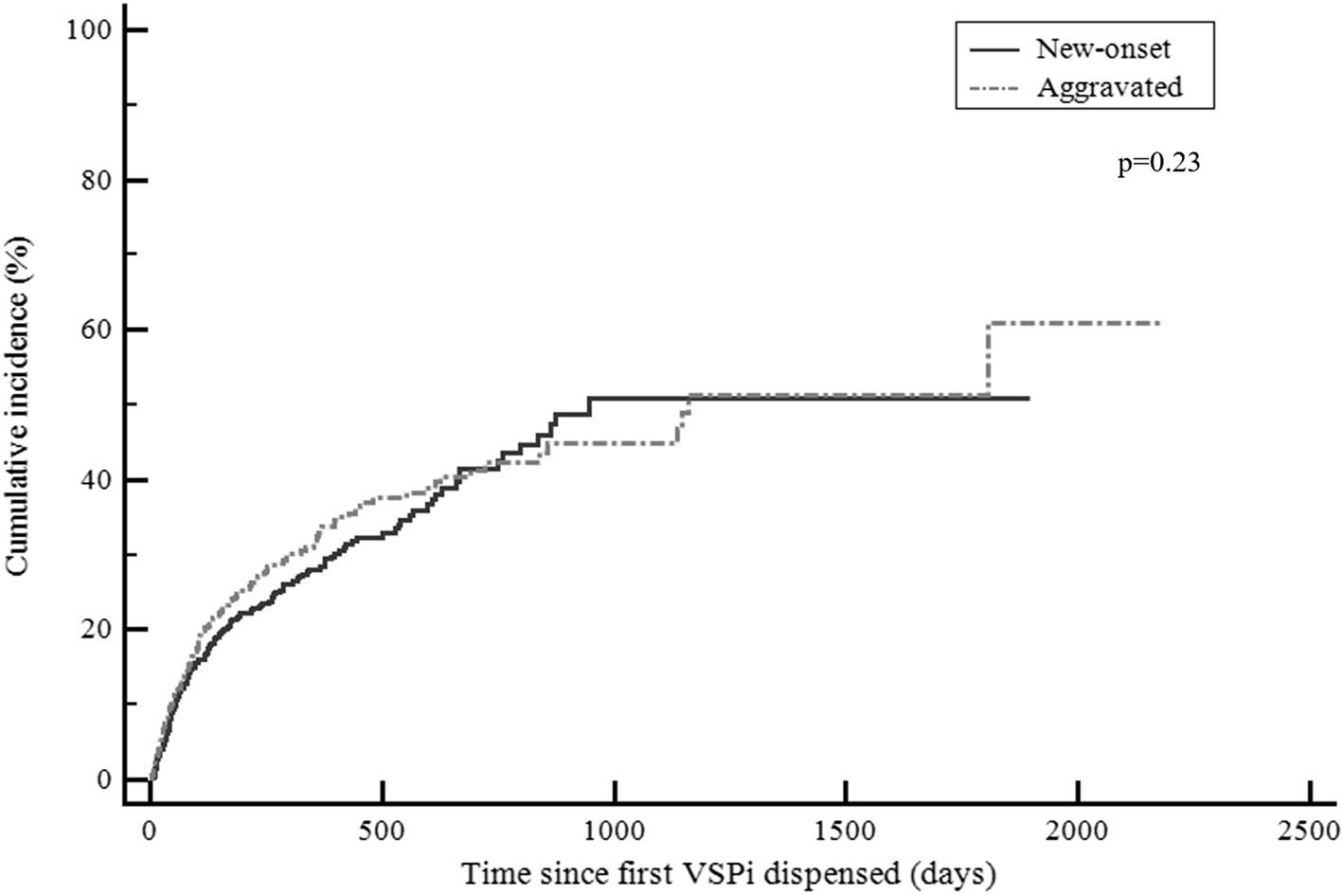
Cumulative incidence of hypertension therapy in Australians dispensed VSPi

The cumulative incidence of new onset and aggravated hypertension over 1 year was not statistically different (Fig. 2).

### Hypertension risk factors

In univariable analyses, age ≥ 60 years, cancer type other than CRC, and oral TKIs were significantly associated with new-onset hypertension. In multivariable analysis, age > 60 years (HR, 1.74; 95%CI, 1.32–2.31) and treatment with oral TKI agents (HR, 1.96; 95%CI, 1.16–3.31) were independent risk factors (Table 3).

**Table 3:** Risk factors for new-onset and aggravated hypertension after VSPi in multivariable analysis

Characteristic	New onset hypertension		Aggravated hypertension	
	HR	95% CI	HR	95% CI
Age > 60 years	1.74	1.32–2.31	1.26	0.86–1.84
Female sex	1.10	0.82–1.48	1.09	0.82–1.44
Higher than median number of comorbid conditions ^a^	0.97	0.74–1.28	0.87	0.67–1.13
Cancer type				
RCC (vs. CRC)	1.22	0.66–2.27	2.84	1.49–5.41
Others (vs. CRC)	1.36	0.87–2.12	1.85	1.12–3.04
Oral TKI (vs. Bevacizumab)	1.96	1.16–3.31	0.68	0.39–1.19

*CRC* colorectal cancer, *HR* hazard ratio, *RCC* renal cell carcinoma, *TKI* tyrosine kinase inhibitor

^a^The median number of comorbid conditions was 3.0

Risk of aggravated hypertension was associated with cancer type other than CRC, and oral TKIs in univariable analyses. In multivariable analysis, only cancer type was an independent risk factor; compared to CRC, risk was elevated for RCC (HR, 2.84; 95%CI, 1.49–5.41) and other cancer types (HR 1.85; 95%CI, 1.12–3.03) (Table 3).

## Discussion

In our real-world study, the overall incidence of new-onset and aggravated hypertension during VSPi treatment was similar, at 24.3% and 25.2% respectively, for a combined overall incidence of 49.5%. These estimates are lower than reported in other observational studies and slightly higher than meta-estimates summarising the clinical trial experience where hypertension was measured [10–12, 23–25]. In clinical trial meta-analyses, the incidence of VSPi-induced hypertension (any grade) was 23.6% for bevacizumab [10], 23.1% for sorafenib [23], and 21.6% for sunitinib [11]. Cancer patients participating in randomized clinical trials are generally highly selected and in otherwise good health, and thus adverse events are observed less frequently than in real-world practice [26]. The two previous US claims-based studies, using International Classification of Diseases (ICD) codes for hypertension or codes for dispensed anti-hypertensive medicines [14, 15] observed incidence estimates of 32.0% for new-onset hypertension and 49.7% for combined new-onset or aggravated hypertension respectively. Our lower estimates for new-onset hypertension may reflect the fact that we could only identify patients dispensed antihypertensives, not all those diagnosed with the condition. In one US real-world clinical practice study, 14.1% of patients with newly diagnosed hypertension during VSPi use were not treated with antihypertensives [14]. In a recent large pooled cohort study of Australian and New Zealand adults who were diagnosed with cancer during follow-up, 33% had untreated hypertension and 25% had treated hypertension at baseline [27]. Hypertension was ascertained via blood pressure measurements and treatment was based on self-report.

For patients with new-onset hypertension, the first antihypertensive dispensing was a median of 78 days from the index date, similar to a previous US claims-based analysis (96 days) [14]. The time interval to hypertension treatment differed by type of VSPi, and was significantly longer for patients treated with bevacizumab than oral TKIs. Among the oral TKIs, the shortest time interval was observed with pazopanib. VSPi-induced hypertension has been shown to develop rapidly, and to return to baseline after drug withdrawal [4, 28, 29]. Studies monitoring blood pressure have revealed blood pressure increases within hours to days after initiating oral TKIs [28, 29]. In one such study, the median time to first documented hypertensive response was 29 days in VSP-TKI treated patients [15].

For patients with new-onset VSPi-induced hypertension, the current guidelines recommend medications targeting the renin–angiotensin–aldosterone (RAAS) pathway such as ACE inhibitors, ARBs, and dihydropyridine CCBs as first-line therapy [30–32]. Most of the patients in our new-onset hypertension group were dispensed ACE inhibitors, ARBs, and CCBs and half started with monotherapy.

We identified patients with pre-existing hypertension based on the dispensing of an antihypertensive agent during the 6 months prior to starting VSPi treatment. Over 50% of our cohort met this criteria, consistent with US claims data [14]. These patients were dispensed an average of two antihypertensive medicines, with ACE inhibitors/ARBs most frequently dispensed. One quarter of our cohort who developed aggravated hypertension received dose intensification and these patients were dispensed an average of three antihypertensive agents after aggravation. Patients experiencing aggravated hypertension were predominantly dispensed ACE inhibitors, ARBs, and CCBs at baseline and their use of diuretics increased after aggravation. In US clinical data, a greater proportion of patients with pre-existing hypertension developed a hypertensive response (55% vs 40%) and pre-existing hypertension was a risk factor for VSPi-induced hypertension [15]. However, we found no significant difference between these two patient subgroups in our study, indicating an index of suspicion is warranted regardless of blood pressure history.

Consistent with US data [15], older age (≥ 60 years) and use of an oral TKI agent compared to bevacizumab were risk factors for new-onset hypertension after VSPi use. Only cancer type was associated with aggravation of hypertension in our cohort, with RCC patients displaying the greatest risk. This is consistent with prior evidence [11, 33, 34], and may be due to higher VEGF levels in RCC compared to other cancer patients, or it may be related to prior nephrectomy or reduced renal function [11, 34]. Alternatively, this may reflect information bias because we could only observe treated hypertension and those with RCC may be more likely to receive antihypertensive therapy.

The major limitation of our study is the lack of clinical information in the PBS database, noting that some antihypertensive medicines are prescribed for other indications. Thus, our data may over-estimate the incidence of clinically treated hypertension. We also could not describe cancer stage, related clinical and laboratory data, or detailed blood pressure measures, nor could we conduct risk factor analyses incorporating these factors. We did not have diagnosis codes and ascertained hypertension through dispensing records. We did not identify an association with baseline comorbidity, potentially because we used the Rx-risk index rather than a weighted index based on diagnostic records. We cannot exclude residual confounding by comorbidity and unmeasured confounders in our multivariable models. PBS records do not contain data for medicines dispensed to public hospital inpatients and we only observed anti-hypertensive medications dispensed in the community, so our study may over-estimate the time to first dispensed anti-hypertensive medication. Despite these limitations, our study covers the entire Australian population and both small molecule TKIs and antibody VSPi.

Our study provides real-world estimates of the incidence of, and risk factors for, VSPi-induced hypertension in a whole-of-population setting. Despite the well-known risks of cardiac adverse events associated with VSPi, there is little existing real-world evidence on hypertension incidence, risk factors and management in VSPi-treated patients. Our findings suggest that the real-world incidence of VSPi-induced hypertension is similar to that observed in pivotal clinical trials and they add valuable data for patients receiving care in routine clinical practice. Australian antihypertensive prescribing data thus appears to be a reliable and cost-effective proxy for clinically identified hypertension in this at-risk population.

## Data Availability

The datasets used in this study are not available for sharing, however, the data can be requested from Services Australia.
